# Gene expression and pollen performance indicate altered postmating selection between *Solanum* species with different mating systems

**DOI:** 10.1093/g3journal/jkaf107

**Published:** 2025-05-15

**Authors:** Timothy J Biewer-Heisler, Matthew J S Gibson, Emily Sornay, Leonie C Moyle

**Affiliations:** Department of Biology, Indiana University, 1001 E 3rd St Room 325, Bloomington, IN 47405, USA; Department of Biology, Indiana University, 1001 E 3rd St Room 325, Bloomington, IN 47405, USA; Department of Biology, Indiana University, 1001 E 3rd St Room 325, Bloomington, IN 47405, USA; Department of Biology, Indiana University, 1001 E 3rd St Room 325, Bloomington, IN 47405, USA

**Keywords:** pollen–pistil interactions, gene expression, selection, cryptic female choice, postmating prezygotic, mating system, pollen tube growth rate, *Solanum*

## Abstract

Postmating prezygotic (PMPZ) traits play an important role in mating success, especially in species where gametes from multiple males compete. Despite this, the effect of mating system transitions, and attendant shifts in the intensity of sexual selection, on specific PMPZ traits and loci is still poorly understood. Here, we assessed differences in pollen PMPZ traits and tissue-specific gene expression (in leaf, pollen, and style) between 2 closely related plant species with different mating systems—*Solanum lycopersicum* (selfing) and *Solanum pennellii* (outcrossing). We focused on species differences in loci with known roles in pollen tube growth rate, including pectin methylesterases (PMEs) and their inhibitors (PMEIs), and in vitro and in vivo pollen tube growth rates. Among the gene expression differences observed between species, we found that the expression domain of pollen-biased genes was much narrower in the selfing species *S. lycopersicum* compared with the outcrossing species *S. pennellii*, including for most reproductive PMEs and PMEIs. In addition, *S. pennellii* had faster pollen tube growth rates in vivo, while *S. lycopersicum* had faster in vitro pollen tube growth rates. We propose that the lower expression of pollen tube development genes in *S. lycopersicum* style tissue, and reduced in vivo pollen performance, is a result of reduced allocation to stylar mechanisms that modulate pollen tube growth, potentially consistent with relaxed selection on cryptic female choice in the selfing species.

## Introduction

Postmating prezygotic (PMPZ) traits, which act after the delivery of male gametes and before fertilization, are critical for successful reproduction. They are also expected to be influenced by sexual selection when there is competition among genotypes for mating, with consequences for both the expression of competitive phenotypes and for the genes that underlie them (Andersson and Iwasa 1996; Bernasconi *et al*. 2004). While these expectations of PMPZ traits have been examined broadly in animal systems (Wigby and Chapman 2004; Firman *et al*. 2017; Garlovsky *et al*. 2024), they remain surprisingly poorly explored in other sexually reproducing groups, including plants (Tonnabel *et al*. 2021). In flowering plants, PMPZ stages of reproduction involve direct interactions between the pollen (the “male” tissue) and the pistil (the “female” tissues that include the stigma on which pollen lands and germinates, and the style through which pollen tubes grow to reach the ovary). At these PMPZ stages, the success of different pollen genotypes can be influenced by both male–male pollen competition and male–female reproductive interactions (Tonnabel *et al*. 2021); the latter include female-mediated mechanisms that bias male gamete success, known as “cryptic female choice” (Eberhard 1996; Firman *et al*. 2017). Evidence supporting the action of strong selection on plant PMPZ traits comes primarily from studies of molecular evolution in genes expressed in pollen, pistils, or both tissues (Clark *et al*. 2006; Moyle *et al*. 2021). However, comparatively little attention has focused on how changing the intensity or nature of selection on PMPZ traits differentially affects the evolution of loci associated with male–male gamete competition, cryptic female choice, or both. Here, our goal is to investigate PMPZ traits and loci to assess evidence for these differential effects.

One broad mechanism that could change the focus or intensity of selection on PMPZ traits is a change in the mating system. Mating system shifts—primarily from outcrossing to selfing—are among the most common reproductive transitions in plants (Barrett 2002). Because they affect the number and identity of mating partners, these shifts are predicted to change selection on PMPZ traits, including those involved in pollen competition and cryptic female choice (Mazer *et al*. 2010). In comparison with outcrossing, where pollen can be received from multiple individuals, selfing often limits male–male competition and female choice to the variation present within 1 individual (Cutter 2019 ). Under these conditions, traits that mediate pollen competition are expected to experience relaxed selection or, if sufficiently costly, to be selected against (Sicard and Lenhard 2011; Wozniak and Sicard 2018; Cutter 2019). Consistent with this, analyses of floral tissues have documented changes in gene expression (Slotte *et al*. 2013; Frazee *et al*. 2021; Z. Zhang *et al*. 2022) and molecular evolution (Moyle *et al*. 2021) accompanying shifts from outcrossing to selfing. However, because few studies investigate molecular differences in individual PMPZ reproductive tissues (although see Pease *et al*. 2016a; İltaş *et al*. 2024), the association between these broad molecular patterns and changes in specific PMPZ traits and genes remains largely correlative.

Pollen tube growth rate is one such trait that mediates PMPZ interactions (Lankinen and Karlsson Green 2015) and could be affected by mating system shifts (Mazer *et al*. 2010). Because only a fraction of growing pollen tubes succeed in fertilizing available ovules within a single flower (Delph and Havens 1998), pollen competition among different genotypes can produce strong selection for faster pollen tube growth (Snow and Spira 1991; Delph *et al*. 1998; Pannell and Labouche 2013). Moreover, factors affecting pollen tube growth rate could also be affected by male–female PMPZ sexual selection (Lankinen and Karlsson Green 2015), where females exert genotype-specific effects on male PMPZ siring success (Marshall and Folsom 1991; Sanchez *et al*. 2004; Fitz Gerald *et al*. 2014; Lu *et al*. 2020; Lubini *et al*. 2023). This male–male and male–female PMPZ selection on pollen growth rate is expected to be relaxed under increased selfing (Mazer *et al*. 2010; Cutter 2019) so that, for example, selfing lineages have slower pollen tube growth rates compared with closely related outcrossing species (e.g. Mazer *et al*. 2018).

Pollen tube growth is also a trait whose molecular basis has been examined in model systems (Zheng *et al*. 2018). Among the contributing molecular factors, pectin methylesterases (PMEs) and their inhibitors [pectin methylesterase inhibitors (PMEIs)] are proteins with known phenotypic effects on pollen tube growth. PMEs and PMEIs influence the flexibility of pollen tube tips and shafts, modifying the degree of tissue calcification based on their relative concentration (Catoire *et al*. 1998; Bosch *et al*. 2005; Jiang *et al*. 2005). Moreover, since PMEs and PMEIs are expressed by both the style and the growing pollen tube (Bosch *et al*. 2005; Pelloux *et al*. 2007; Ezura *et al*. 2017; G. Y. Zhang *et al*. 2010), their influence on pollen tube growth rate depends on the combined expression of PMEs and PMEIs from both tissues. The joint ability of PMEs and PMEIs to influence pollen tube growth rate, via expression in both male and female tissues, makes them an interesting lens through which to study the contributions of male and female tissues to differential pollen performance traits between species with different mating systems.

In this study, we sought to assess how loci associated with pollen tube growth (and thereby, potentially, with male–male gamete competition and cryptic female choice) are affected by a change in mating system, focusing specifically on the expression of PME and PMEI genes. To do so, we re-examined reproductive (pollen and style) and nonreproductive (leaf) gene expression data from 2 species in the wild tomato group—*Solanum* section *Lycopersicon*—that differ in their predominant mating system. In addition to quantifying global patterns of gene expression variation and tissue-bias, we identified reproductive-specific PME and PMEI loci and the distribution of their expression in male (pollen) and female (pistil) tissues in each species. To better interpret the relevance of detected gene expression differences for pollen performance within each species, we also quantified their pollen phenotypes in vivo (growing within a female pistil/reproductive tract) and in vitro [growing independent of the female pistil, in pollen growth media (PGM)]. Combining our observations of reproductive PME/PMEI expression with pollen tube growth rates, we develop 1 possible model that connects shifts in gene expression to changes in selection on male- and female-side PMPZ traits that accompany mating system changes.

## Materials and methods

### Study species

Our analysis focused on 2 species in the wild tomato clade (*Solanum* section *Lycopersicum*) that vary in their mating system: *S. lycopersicum* (domesticated tomato; genotype LA3475) and *S. pennellii* (genotype LA0716). Species in this clade range from obligately outcrossing (via gametophytic self-incompatibility) through to primarily selfing (Bedinger *et al*. 2011). *S. lycopersicum* is genetically self-compatible and frequently self-fertilizes. *S. pennellii* is a wild tomato species that is historically genetically self-incompatible (Covey *et al.* 2010a). The specific genotype of S. pennellii in our experiment has recently transitioned to self-compatibility via loss-of-function mutations in the self-incompatibility mechanism (Covey *et al.* 2010a) and has had a history of ex-situ cultivation since the last century (Rick and Tanksley 1981). As a result, it has reduced heterozygosity compared with other outbred (self-incompatible) accessions of *S. pennellii* but remains less homozygous than historically self-compatible and inbreeding species of *Solanum* (see, for example, Fig. 3 in Pease *et al*. (2016b)). Our accessions of *S. pennellii* and *S. lycopersicum* also differ in floral traits consistent with their inferred differences in outcrossing rate (Peralta and Spooner 2005; Bedinger *et al*. 2011). *S. lycopersicum* flowers are smaller, with shorter styles and inserted stigmas that promote self-fertilization; *S. pennellii* flowers are significantly larger, with a stigma and style that are exerted beyond the anthers, consistent with outcrossing (Vosters *et al*. 2014). A previous study of post-pollination isolating barriers between these 2 species (Pease *et al*. 2016a ) analyzed both pollen and stigma/style gene expression and found substantial global transcriptomic differences between them.

### RNA-Seq data and gene expression quantification

Gene expression (RNA-seq) data were obtained for leaf, pollen, and stigma/style tissues from 2 published datasets (PRJNA245845 from Ichihashi *et al*. (2014) and PRJNA309342 from Pease *et al*. (2016a)). The leaf dataset (from Ichihashi *et al*. (2014)) includes samples from 4 early leaf development stages (P3-P6 primordia) and 2 spatial positions within 3 of these stages (distal vs proximal samples), across 3 biological replicates. Because our goal was to use general patterns of gene expression in leaf tissue as a contrast for reproductive (pollen and style) tissue expression, we used the average gene expression across all 7 subtypes of leaf tissue within each biological replicate. The pollen and style data were from Pease *et al.* (2016a), which contains details of their collection. The pollen dataset included 2 categories, ungerminated and germinated pollen; for our analyses, we used the average gene expression across these 2 subcategories within each biological replicate. The style dataset also included 2 categories of mature style, unpollinated and pollinated styles; however, because pollinated styles represent a mixed tissue (both style and pollen), we only included data from the “mature unpollinated style” (Pease *et al*. 2016a) tissue in our analysis. To generate this tissue, flowers were emasculated 24 h prior to anthesis (opening) to prevent precocious pollination and to ensure that reproductively mature style tissue collected 24 h later did not inadvertently include pollen (see Pease *et al*. 2016a). For simplicity, we refer to this tissue as “style”, although collected styles included intact stigmas, so these data technically encompass both style and stigma gene expression. For both pollen and style data, we had 3 biological replicates.

For each of the above tissues, we used the FASTQ preprocessor fastp (Chen *et al*. 2018) to preprocess and quality-filter the archived raw read data. Only reads >15 bp were retained; in addition, at least 60% of bases in each read were required to have Phred quality scores greater than or equal to 25. Cleaned reads were mapped to the tomato reference genome (SL4.0) using the annotation-aware read mapper STAR (Dobin *et al*. 2013). Tomato genome annotation ITAG4.0 was used to create the genome index used by STAR. Read counts were estimated with featureCounts (Liao *et al*. 2014). Overall, all 3 tissue types had high total sequencing depth (>15×) postfiltering, indicating similar power to detect gene expression (see Supplementary Text 1).

Raw read counts were normalized in 2 ways, depending on the target analysis. First, analyses depending on tissue-specificity and -bias require normalization by TPM (transcripts per million) (see next section). Therefore, we applied the standard TPM normalization method, which involves determining the reads per kilobase for each gene and dividing that by the scaling factor in R. Only loci with an average TPM ≥ 2 (across biological replicates) in at least 1 tissue and at least 1 species were included in the associated analyses. This resulted in a total dataset of 21,039 expressed loci. Second, differential expression analyses of individual loci in the *DESeq2* package (Love *et al*. 2014) (as described below) require that normalized gene expression is quantified as the median of ratios; this normalization was performed within *DESeq2*.

### Determining tissue-specificity and bias

We classified gene expression among tissues within species in terms of 2 classes of gene expression: “tissue-specific” and “tissue-biased” genes. “Tissue-specific” genes were defined within each species dataset as those expressed solely in one tissue type (i.e. leaf, pollen, or style) and not in any other tissue (cutoffs for expression are specified below). In comparison, “tissue-biased” genes were defined within each species dataset as genes whose expression was enriched in one tissue type (i.e. leaf, pollen, or style) compared with the other tissue types (as determined by the metric Tau, below). Accordingly, tissue-specific genes are a subset of tissue-biased genes. In addition, in each of our species, we also classified loci according to whether they were specific to nonreproductive or reproductive tissues (i.e. expressed in leaf tissue only, or pollen and/or style only, respectively).

Tissue-specificity and -bias were determined for each gene within each species dataset using the metric tau (Yanai *et al*. 2005):


$$\tau =\frac{\Sigma_{i=1}^{N}(1-\hat{x_{i}})}{N-1};\;\;\hat{x_{i}}=\frac{x_{i}}{\max(x_{i})}$$

where *N* is the number of tissues and $\hat{x_{i}}$ is the gene expression normalized by the maximal tissue expression of that gene. Loci with tau = 1 are tissue-specific. Loci with a tau value greater than or equal to 0.7 were designated as biased toward the tissue in which they were most highly expressed. This threshold for tissue-bias is common in the literature, including in studies with a similar number of tissue comparisons (Osterwalder *et al*. 2018; Jansen *et al*. 2021; McDonough-Goldstein *et al*. 2021), and represents a reasonable balance between identifying loci whose expression is substantively enriched in one tissue only, without being so stringent as to exclude genes whose expression is primarily but not exclusively observed in this class of tissue. Our major inferences are also robust to changes in this threshold (Supplementary Text 1). All other genes were categorized as unbiased.

### Global patterns of gene expression variation between species

To compare broad patterns of gene expression level and tissue-specificity between species, we used linear models with species as a factor. These models assessed whether there were differences between species in the average magnitude of pollen, style, or leaf expression bias (i.e. the mean tau for each tissue), or in the average level of gene expression (TPM) in each of these classes of locus.

To evaluate functional classes of genes expressed within each species, genes designated as pollen, style, and leaf biased in each species were assessed for functional overrepresentation by performing a Gene Ontology (GO) term enrichment analysis in *Panther* v17 (Mi *et al*. 2019; Thomas *et al*. 2022). Each group of tissue-biased loci within each species was compared against the list of all expressed genes in our dataset. These analyses assessed enrichment in 3 different classifications of functional category: biological process, molecular function, and protein class. Significance was determined using Fisher's exact tests with Bonferroni correction for multiple testing. All statistics were calculated using the PANTHER Overrepresentation Tests.

### Differential expression of individual loci between species

To identify which individual genes were differentially expressed between species, specifically in pollen and style tissue, we used the *DESeq2* package (Love *et al*. 2014) in R. For each locus, normalized gene expression in each sample was quantified as the median of ratios, as required in *DESeq2*. A generalized linear model was fit to each gene in our dataset of tomato loci (*N* = 33,562), in which expression counts were modeled as a function of species and tissue. Replicate samples were nested within each tissue category (leaf, pollen, and style). Of all genes, *N* = 29,999 loci were expressed at any level in any tissue (Supplementary Table 1). In addition, for the subset of these loci whose expression was biologically significant (i.e. >2 TPM in any tissue) (*N* = 21,039), we performed 2 planned contrasts (Supplementary Table 1), specifically: pollen expression between species and style expression between species. The Benjamini–Hochberg method for multiple test correction (Benjamini and Hochberg 1995) was applied for each contrast performed (2 contrasts for each locus evaluated; postcorrection *P* < 0.05).

To explore functional roles of highly differentially expressed reproductive (pollen or style) genes specifically, we identified the 10 loci with the largest differences (TPM) between species in each of 4 categories: pollen-specific, style-specific, pollen-biased, and style-biased (Supplementary Table 2). Gene protein functions for these loci were evaluated based on functional annotations on the Sol Genomics Network (Fernandez-Pozo *et al*. 2015). For completeness, similar data for all loci that were differentially expressed between species in pollen or style tissue are reported in Supplementary Table 3.

### Identifying PME and PMEI genes in the tomato genome

PMEs and PMEIs were identified by searching the tomato annotation ITAG4.0 for functional labels consistent with these 2 gene families (see Supplementary Table 4 for specific terms). This search generated 76 PMEs and 32 PMEIs. For these genes specifically, we evaluated tissue-specificity and tissue-bias in each species (Supplementary Tables 5 and 6), as well as patterns of gene expression variation among tissues and among species (Supplementary Tables 7 and 8), using the same methods as applied to the larger dataset. For completeness, we also evaluated the same patterns for 5 loci with previously described functions in *Solanum* pollen–pistil interactions between species, especially related to the expression of interspecific unilateral PMPZ incompatibility (Li and Chetelat 2010, 2014; Tovar-Mendez *et al*. 2014, 2017; Qin *et al*. 2018; Muñoz-Sanz *et al*. 2021; Qin and Chetelat 2021) (Supplementary Table 9 and Text 1).

### Assays of pollen tube growth phenotypes

To assess rates of pollen tube growth, we performed in vitro and in vivo pollen germination and growth assays in 5 biological replicates (pollen donors) per species. We collected pollen from plants grown in the Indiana University Bloomington greenhouses, across several months (November 2022–May 2023 for in vitro assays; May 2023–August 2023 for in vivo assays). Pollen was collected between 8 Am and 12 Pm via artificial vibration of floral anther cones. Pollen for in vivo assays was collected fresh on the day of each assay and held in 0.2 ml microcentrifuge tubes before being used. Pollen for in vitro assays was collected and stored in 0.2 ml microcentrifuge tubes at −20°C until use. Freezing reportedly does not affect the fertility of *Solanum* pollen, even when frozen for up to 12 months (Song and Tachibana 2007). We have also observed that the qualitative in vitro PT performance of *S. lycopersicum* and *S. pennellii* does not differ between fresh and frozen pollen (Jewell 2016 and unpublished data), and that species differences in in vitro performance are consistent across multiple timepoints postgermination (Supplementary Table 10). Nonetheless, we evaluated a subset of individuals in the current study, using unfrozen pollen in in vitro assays, to confirm that any qualitative differences between fresh and previously frozen pollen did not affect our comparisons between species. We did detect absolute differences in pollen tube growth rate between unfrozen and previously frozen pollen (unfrozen pollen generally grew faster in both species; Supplementary Table 10) but confirmed that the qualitative difference and relative rank of species performance in in vitro assays was the same regardless of whether unfrozen and previously frozen pollen was used (see *Results*). Before use in the in vitro assays, all pollen collections from each biological replicate individual were aggregated, vortexed, and aliquoted into subsamples for use as technical replicates.

In vitro assay: To assess pollen germination and growth in the absence of stylar tissue, sampled pollen was germinated on PGM of 2% sucrose, 24% PEG-4000, 0.01% borate, 20 mM HEPES, 3 mM calcium nitrate, 0.02% magnesium sulfate, and 0.01% potassium nitrate (Covey *et al*. 2010b). For each assay, each pollen sample was mixed with 50 μl of PGM and placed as a droplet in 1 well of a 24-well cell culture plate. A sample from every biological replicate (5 plants per species × 2 species =10) was included in each plate; the entire assay was repeated 5 times (5 technical replicates) in May 2023. Each replicate plate was cultured in the dark for 3 h at 22.4°C. After 3 h, each sample was imaged at 10× magnification (including a standard scale bar) on an AMG EVOS Fl microscope.

We used ImageJ to generate the following data from each sample image: the number of viable pollen, the number of germinated pollen, the average diameter of pollen, and the average length of pollen tubes. Pollen viability was assessed via shape; collapsed elliptic pollen grains were classified as inviable, and round semi-transparent pollen grains were classified as viable. In each image, pollen diameter and pollen tube growth were measured on 10 randomly sampled germinated pollen by overlaying an 8 × 11 grid and measuring the germinated pollen that was closest to each of 10 predetermined grid locations. Values for each image (sample) were averaged across the 10 randomly sampled pollen. Only viable pollen was included in these metrics. Technical replicates were averaged to give a single value for each biological replicate, prior to analysis.

In vivo assay: To assess pollen germination and growth within stylar tissue, each of 5 pollen donor individuals was assayed on pistils of 3 additional biological individuals (pollen recipients) from their own species. Within each species, every specific combination of pollen donor and recipient (5 donors × 3 recipients = 15 unique combinations) was repeated at least twice. Recipient plants were grown in the Indiana University Bloomington greenhouses, and assays were performed between May and August 2023. For each assay, unopened flowers on recipient plants were emasculated 24 h prior to the assay by removing the anther cone using forceps. On the following day, pollen was collected from each available (flowering) pollen donor; each emasculated style received the pollen of a single donor from their species. Pollinations were performed by coating the stigma with collected pollen held in the cap of a 0.2 ml microcentrifuge tube. All pollinations were performed between 8 Am and 12 Pm. Each pollinated pistil was collected 5–7 h after pollination by detaching it at the point where the style meets the ovary. Collected pistils were stored in 200 μl of 25% acetic acid fixing solution at −20°C.

Aniline blue staining was used to visualize pollen tubes within each collected pistil. For each collected sample, the fixing solution was replaced with 200 μl 5 M NaOH and held for 24 h at room temperature in the dark. After 24 h, the 5 M NaOH was removed, the pistil washed with 200 μl 0.1 M K_3_PO_4_ and stained with 200 μl 0.01 mg/ml aniline blue, 0.06 M K_3_PO_4_. After staining for 5–8 h, each pistil was imaged at 10× magnification (including a standard scale bar) under UV light on an AMG EVOS Fl microscope. Each sample required multiple images to capture the complete length of the style. Images of the same style were stitched together in Adobe Photoshop prior to collecting phenotypic data in ImageJ. From each stitched image, we measured style length in addition to the average pollen tube length of the 5 longest pollen tubes. We have previously found that this and other metrics of PT performance—including the distance traveled by the single fastest pollen tube or by the majority of pollen tubes (the PT “front”)—are strongly correlated within species (e.g. Jewell 2016; Jewell *et al*. 2020).

To analyze PMPZ phenotypes, we used linear models (*T*-tests and ANOVAs) in the *stats* package in R. *T*-tests were used to assess species differences in average germination rate, pollen tube growth rate, and pollen diameter (in in vitro assays), and average style length, pollen tube growth rate, and proportion of style traveled (in in vivo assays). We used follow-up ANOVAs (on pollen diameter and in vitro pollen tube growth rates) to also assess evidence for differences among individuals within each species, and (for in vivo growth rates) the effect of assay date. To compare the variance of in vitro pollen tube growth rates between species, we used an *F*-test (also called a Bartlett test, or, in the *stats* package, a variance test) with species as a factor.

## Results

### Species express similar numbers of genes, but differ in the proportions of loci that are specific to pollen

**Fig. 1.**
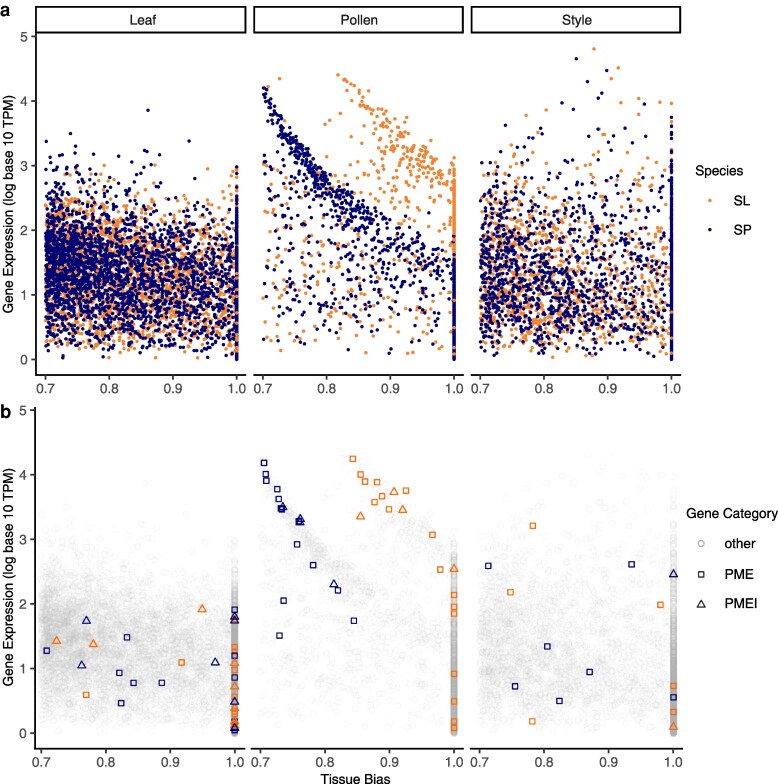
a) Gene expression in all leaf-biased (left), pollen-biased (middle), and style-biased (right) genes, in *S. lycopersicum* (SL, orange) and *S. pennellii* (SP, blue). Each point is an individual gene. Gene expression is normalized (in log_10_ TPM units) and tissue-bias is in units of tau. See Table 1 for *N* in each category. The average tau of *S. lycopersicum* pollen-biased genes is significantly higher than the average tau of *S. pennellii* pollen-biased genes (linear model on tau between species: *P* < 2e-16; Supplementary Table 8). b) Gene expression of PMEs and PMEIs among all leaf-biased (left), pollen-biased (middle), and style-biased (right) genes, in each species. PMEs are squares, PMEIs are triangles, and non-PME/PMEIs are circles (and transparent gray).

Species expressed similar numbers of total genes (∼19,600; Table 1), and most (>80%) expressed genes were detected in both style and leaf tissues in both species (Supplementary Table 11). For all 3 tissues, the fraction of genes classified as tissue-biased was also broadly equivalent between the species (Table 1), as was the proportion of style- and leaf-biased genes that were tissue-specific (i.e. loci with tau = 1, Table 1). In contrast, species differed substantially in the proportion of pollen-biased genes that were pollen-specific: *S. lycopersicum* had nearly twice as many pollen-specific genes (631 of 1,132 pollen-biased genes) as was observed in *S. pennellii* (340 of 1,027 pollen-biased genes; Table 1; Fig. 1a). Consistent with this, the average magnitude of pollen-bias (tau) was significantly higher in *S. lycopersicum* (linear model on all pollen-biased genes between species: *P* < 2e-16; Supplementary Table 12).

**Table 1.** The number of genes expressed at >2 TPM in each tissue, in each species.

Gene types	SL Total	SL Pollen	SL Style	SL Leaf	SP Total	SP Pollen	SP Style	SP Leaf
Expressed genes	19,667	6,143	16,200	17,418	19,589	6,030	16,315	17,234
Biased genes	6,656	1,132	1,711	3,813	7,055	1,027	1,873	4,155
*^a^* Prop. expressed that are biased	0.338	0.184	0.106	0.219	0.360	0.170	0.115	0.241
Specific genes	2,918	681	645	1,592	2,882	340	760	1,782
*^b^* Prop. expressed that are specific	0.438	0.602	0.377	0.418	0.408	0.331	0.406	0.429

SL , *S. lycopersicum*; SP, *S. pennellii*.

^*a*^Within a tissue, the proportion of expressed genes whose expression is biased toward that tissue.

^*b*^Within a tissue, the proportion of tissue-biased genes that were tissue-specific.

### Fewer genes are pollen-biased or -specific in the outcrossing species, reflecting higher secondary expression of these loci in style tissue

**Fig. 2.**
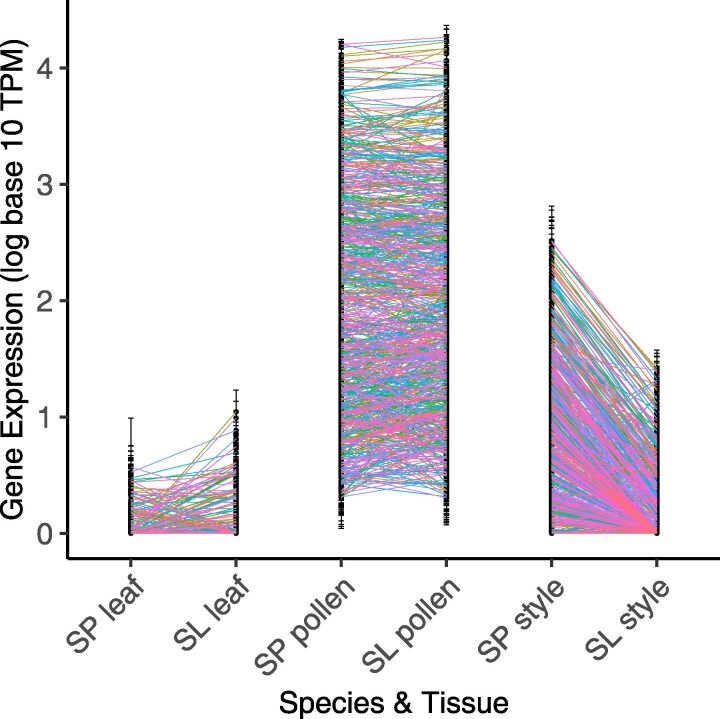
Gene expression in all pollen-biased genes (loci that were pollen-biased in at least 1 species) with TPM >2 in pollen, by species and reproductive tissue. Gene expression is normalized (in log_10_ TPM units). Average pollen expression does not differ between species (*P* = 0.754; Supplementary Table 8); style expression does significantly differ (*P* < 2e-16; Supplementary Table 8). SP, *S. pennellii*; SL, *S. lycopersicum*.

The difference in pollen-bias and -specificity between species is not explained by differences in average pollen expression level (TPM) across all pollen-biased genes (linear model on expression between species: *P* = 0.754; Fig. 1a; Supplementary Table 12). Instead, we found that genes are less pollen-biased and -specific in *S. pennellii* because these loci have significantly greater secondary expression in *S. pennellii* styles. That is, *S. pennellii* pollen-biased genes are expressed at higher levels in *S. pennellii* style tissue, compared with the stylar expression of pollen-biased genes in *S. lycopersicum* (linear model on expression between species: *P* < 2e-16; Fig. 2; Supplementary Table 12). Moreover, of the 482 pollen-biased genes that have secondary expression in style tissue in either species, only 11 have higher expression in *S. lycopersicum*, while 471 have higher expression in *S. pennellii* (Supplementary Table 13; Fig. 2). Strikingly, over half of all pollen-biased genes (254 of 482) are not expressed at all in *S. lycopersicum* styles. Overall, the expression domain of pollen-biased genes is much narrower in *S. lycopersicum* compared with *S. pennellii*, a pattern only observed for pollen-biased genes.

In comparison with pollen-biased genes, *S. pennellii*'s leaf-biased genes had a significantly higher average tau than *S. lycopersicum*'s leaf-biased genes (linear model on tau between species: *P* = 0.003; Fig. 1a; Supplementary Table 12). The average tau of style-biased genes did not differ significantly between species (Fig. 1a). This underscores that greater tissue-bias is not a general feature of *S. lycopersicum* but is observed specifically in pollen-biased genes in this species.

### Pectinesterase and pectinesterase inhibitor functions are overrepresented in pollen-biased genes

Compared with all expressed genes in our dataset, our GO analyses found several molecular functions, protein classes, and biological processes were overrepresented in pollen-, style-, and leaf-biased genes in each of *S. lycopersicum* and *S. pennellii* (Supplementary Tables 14–19). In pollen-biased genes, the pectinesterase (GO:0030599) molecular function was significantly overrepresented in both species, and the pectinesterase inhibitor (GO:0046910) molecular function was significantly overrepresented in *S. lycopersicum* (Supplementary Table 15). Other highly overrepresented categories for pollen-biased genes in one or both species included various actin and/or cytoskeletal protein classes (actin or actin-binding cytoskeletal protein (PC00041), cytoskeletal protein (PC00085), and nonmotor actin-binding protein (PC00165), as well as monovalent inorganic cation homeostasis (GO: 0006885) and multiple phospholipid and/or phosphatidylinositol molecular functions (Supplementary Tables 15 and 18) that are known to play an important role in pollen tissue structure (Brewbaker and Kwack 1963) or pollen development (Lee *et al*. 2008) including in *S. lycopersicum* (Huang *et al*. 2014). The categories overrepresented in style-biased genes in both species included oxygenases (PC00177), oxidoreductases (PC00176), and metabolite interconversion enzymes (PC00262) (Supplementary Tables 16 and 19). The categories overrepresented in the leaf-biased gene group for both species (Supplementary Tables 14 and 17) were largely separate from those overrepresented in pollen- or style-biased genes, with the exception of a few general processes.

### Reproductive genes that are highly differentially expressed between species have protein functions involving pollen signaling and development

We determined the annotated functions of the 10 loci with the largest expression difference (TPM) between species in each of the pollen and style tissues, for both tissue-biased and tissue-specific subsets of the data (Supplementary Table 2). These included several loci with known functional roles in pollen tube growth, modification of plant cell walls, or pollen–pistil signaling. In pollen-biased genes, these included a pectin methylesterase gene (Solyc05g054360.4), which showed higher expression in *S. lycopersicum*, and a beta-D-glucosidase gene (Solyc01g009240.4), which showed higher expression in *S. pennellii.* Style-specific genes include the locus DIR1L (Solyc01g109390.3)—previously described to mediate style-side PMPZ interspecific barriers between *Solanum* species (Muñoz-Sanz *et al*. 2021)—which showed significantly higher expression in *S. pennellii* styles. Style-biased genes also included a defensin gene (Solyc07g007730.4), with a GO term for peptidase inhibitor activity that was more highly expressed in *S. lycopersicum*, and a 1-aminocyclopropane-1-carboxylate oxidase (ACC) protein gene (Solyc07g049550.3) that was more highly expressed in *S. pennellii*.

### Most reproductive-specific PMEs and PMEIs have lower style, but not pollen, expression in the selfing species

For the 76 PME and 32 PMEI genes, we inferred in the tomato genome (see *Methods*), we determined the tissue-bias and specificity of these loci (Supplementary Tables 5–8 and 20). Of PMEs and PMEIs expressed in any tissue, many were strongly style- or (especially) pollen-biased, in one or both species (Supplementary Table 20; Fig. 1b). We found 16 of 76 PMEs and 5 of 32 PMEIs were specific to reproductive tissues (i.e. expressed at >2 TPM in only style and/or pollen tissue) (Supplementary Table 20).

**Fig. 3.**
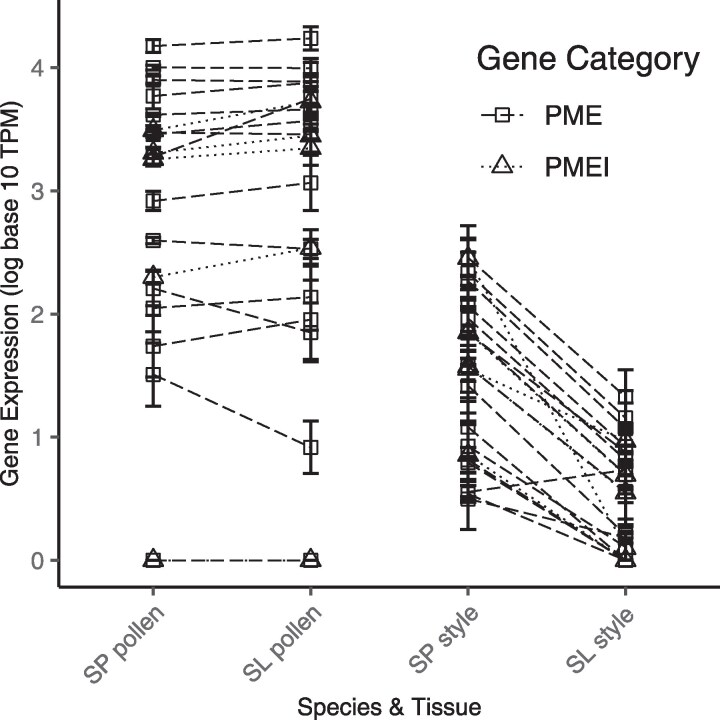
Gene expression for exclusively reproductive PMEs (*N* = 16) and PMEIs (*N* = 5), by species and tissue (Supplementary Tables 4 and 5). PMEs are square, with dashed lines. PMEIs are triangles, with dotted lines. Average pollen gene expression was not significantly different between species (PMEs: *P* = 0.732, PMEIs: *P* = 0.486; Supplementary Table 8). Average style gene expression was significantly different for PMEs (*P* = 0.0053; Supplementary Table 8) but not PMEIs (*P* = 0.136; Supplementary Table 8). Gene expression is normalized (in log_10_ TPM units). SP, *S. pennellii*; SL, *S. lycopersicum*.

Across all 16 reproductive-specific PMEs, the average level of stylar gene expression (TPM) was significantly greater in *S. pennellii* than in *S. lycopersicum* (linear model on expression between species: *P* = 0.0053; Fig. 3). Moreover, 15 of these 16 loci individually showed significantly higher expression (normalized median of ratios) in the style of *S. pennellii* than in the style of *S. lycopersicum* (Supplementary Table 7). The remaining PME is significantly more expressed in *S. lycopersicum* style but is lowly expressed in both species (5.4 vs 3.5 TPM; Supplementary Tables 6 and 7). Each of the 5 reproductive-specific PMEIs was also individually more highly expressed (normalized median of ratios) in *S. pennellii* styles than in *S. lycopersicum* styles (Supplementary Table 8; Fig. 3), despite their overall average expression (TPM) not significantly differing between species (linear model on expression between species: *P* = 0.136; Fig. 3).

In contrast to style expression, average pollen expression (TPM) across all PME or PMEI reproductive-specific genes did not differ between species (linear model on expression between species: PMEs: *P* = 0.732, PMEIs: *P* = 0.486; Fig. 3). Of the 16 reproductive-specific PMEs, one was individually more highly pollen-expressed in *S. lycopersicum*, and one was more highly expressed in *S. pennellii* (Supplementary Table 7). No reproductive-specific PMEIs had significantly different pollen expression between species (Supplementary Table 8).

### Relative pollen tube growth rate differs between species, depending on whether pollen is growing in vitro or in vivo

To better interpret the role of detected gene expression differences in pollen performance traits within each species, we also quantified their pollen phenotypes in the presence (in vivo) and absence (in vitro) of the female style, and therefore with and without pollen–pistil interactions (Supplementary Table 21). Regardless of context, *S. pennellii* has larger pollen grains than *S. lycopersicum* (linear model on species, *P* < 2e-16; Supplementary Table 22), a pattern consistent among biological replicates within species (*P* = 0.123; Supplementary Table 22).

**Fig. 4.**
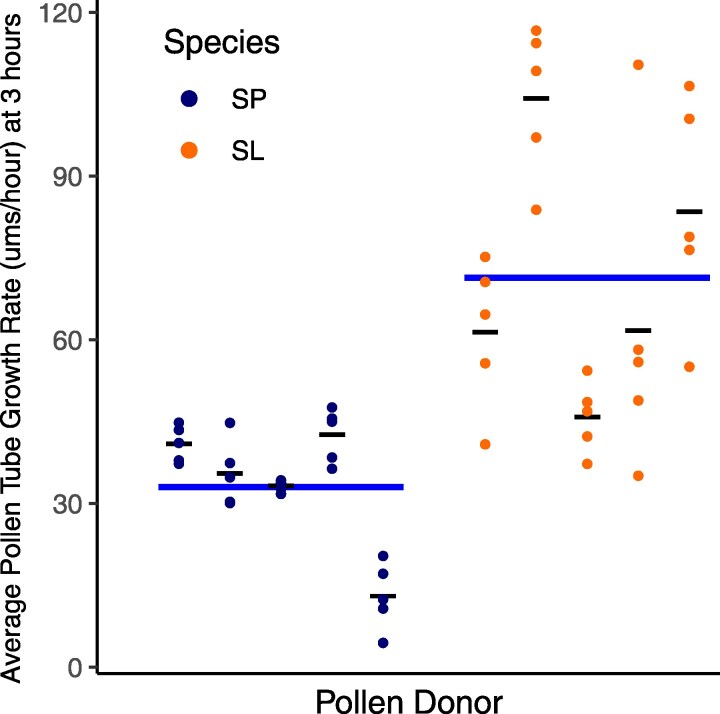
In vitro pollen tube growth rate in μm/h for *S. pennellii* (SP, blue) and *S. lycopersicum* (SL, orange). Average growth rate for each individual is shown as a thin black line. Average growth rate for each species is represented as a thick blue line. *S. lycopersicum* pollen tubes were significantly longer than *S. pennellii* pollen tubes (linear model on species, *P* = 1.18e-12; Supplementary Table 14c).

In our in vitro assays, *S. lycopersicum* pollen had significantly faster pollen tube growth rates on average than *S. pennellii* (Fig. 4, linear model on species, *P* = 1.18e-12; Supplementary Table 22). This in vitro difference between *S. lycopersicum* and *S. pennellii* is observed whether frozen or fresh pollen was used (in our assays of the same experimental plants here; Supplementary Table 21), and across multiple timepoints (from 30 min to 3 h) postgermination (including in prior pilot observations of fresh pollen growth rates; Supplementary Tables 10a and 21). We also detected significant variation in PT growth rate among replicates within species (*P* = 4.05e-07; Supplementary Table 22), which is explained by greater variance among biological replicates (pollen donors) in *S. lycopersicum* compared with *S. pennellii* (Bartlett test on pollen tube growth rate between species, *P* = 0.00013; Supplementary Table 22). Unlike PT growth rates, in vitro pollen germination rates did not significantly differ between species (Supplementary Table 21).

**Fig. 5.**
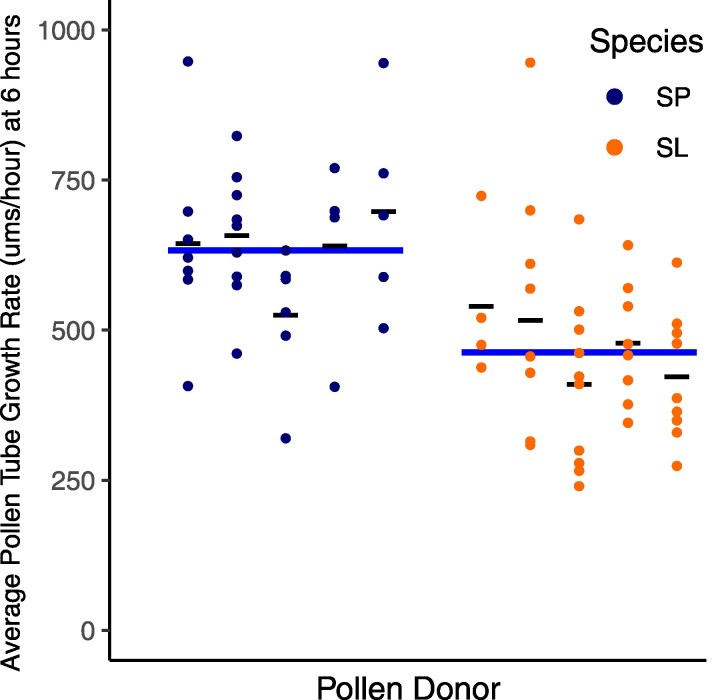
In vivo pollen tube growth rate in μm/h for *S. pennellii* (SP, blue) and *S. lycopersicum* (SL, orange), for each pollen donor. Average growth rate for each individual is represented as a thin black line. Average growth rate for each species is represented as a thick blue line. *S. pennellii* pollen tubes were significantly faster than *S. lycopersicum* pollen tubes (linear model on species, *P* = 0.000318; Supplementary Table 14c).

In contrast to in vitro assays, in in vivo assays we found significantly faster PT growth rates in *S. pennellii* (Fig. 5, linear model on species, *P* = 0.0003; Supplementary Tables 21 and 22). We also detected significant variation in PT growth rate among biological replicates within species (linear model on biological replicates as a factor of species, *P* = 0.0287; Supplementary Table 22), although this could not be ascribed to a specific species. Finally, because *S. pennellii* style lengths are consistently longer (linear model on species, *P* < 2e-16; Supplementary Table 21), species did not differ in the proportion of the style traversed by pollen tubes (linear model on species, *P* = 0.3492; Supplementary Table 21), despite *S. pennellii*'s significantly faster in vivo PT growth rates.

## Discussion

In this study, we investigated differences in pollen, style, and leaf gene expression, particularly for PMEs and PMEIs, between historically outcrossing *S. pennellii* and selfing *S. lycopersicum*. We also observed pollen phenotypes in vivo and in vitro for both species, comparing pollen tube growth rates with and without the presence of the style, respectively. The most striking patterns we detected were a consistent difference between species in whether and how much pollen-biased genes were secondarily expressed in style tissue, as well as consistent species differences in pollen tube growth rates. The outcrossing species, *S. pennellii*, had greater style expression of many pollen-biased genes, including most reproductive PMEs and PMEIs. *S. pennellii* pollen also had faster in vivo growth rates. In contrast, the selfing species *S. lycopersicum* had higher pollen expression of 1 very highly expressed PME and more rapid in vitro pollen tube growth rates. Below we expand on these findings, including proposing 1 model for how this difference in style expression of pollen tube development genes might be due to altered selection on PMPZ traits—including via mechanisms such as cryptic female choice—between outcrossers and selfers.

### Tissue-specific expression patterns identify PMPZ reproductive loci, including PME/PMEIs, that are differentially expressed between species

Our analysis identified the class of genes whose expression was biased or exclusive to 2 reproductive tissue types, enabling us to assess broad functional classes within each tissue, to identify PME and PMEI loci with reproductive-specific expression, and to identify differences between our focal species. Among reproductively expressed loci (genes that are biased or exclusive to pollen and style), we found significant enrichment of several classes of pollen performance and pollen–pistil-related functions. Among pollen-biased genes, in particular, we confirmed that the pectinesterase and pectinesterase inhibitor molecular functions were significantly overrepresented in one or both species—consistent with their known role in pollen tube growth and development. Other overrepresented categories in one or both species are broadly consistent with essential cellular functions in our reproductive tissues (see *Results*).

We also identified the subset of *Solanum* PMEs and PMEIs (16 and 6, respectively) that are reproductive-specific (Supplementary Tables 5 and 6)—and could thereby have potential functions in pollen tube growth modulation. Almost all of these loci also showed significant stylar expression differences between species, suggesting that their roles in the style have diverged between *S. pennellii* and *S. lycopersicum*. In contrast, only 2 PMEs differed in pollen expression between species, although one of these—a pectin methylesterase gene (Solyc05g054360.4) with elevated expression in *S. lycopersicum*—was among the 10 loci with the largest expression differences between species in pollen-biased genes (Supplementary Table 2).

Beyond PMEs and PMEIs, species were also significantly differentiated for loci with other known pollen performance and pollen–pistil functions (Supplementary Tables 2 and 9). Highly differentially expressed style-specific genes included an ACC protein gene (Solyc07g049550.3). ACC proteins have previously been associated with pollen tube directional signaling in *Arabidopsis thaliana* and other systems (Higashiyama 2010; Mou *et al*. 2020), as well as stimulating pollen tube growth in *S. lycopersicum* (Althiab-Almasaud *et al*. 2021) (Supplementary Table 2). Among loci with previously described functional roles in PMPZ interactions between *Solanum* species, the majority were significantly more highly expressed in *S. pennellii* either in pollen or style tissue (Supplementary Table 9) consistent with their previously identified roles in unilateral expression of interspecific incompatibility (Li and Chetelat 2010, 2014; Qin *et al*. 2018; Muñoz-Sanz *et al*. 2021; Qin and Chetelat 2021) (Supplementary Text 1).

Together, these results indicate an enriched expression of known and putative pollen performance and pollen–pistil loci—including multiple PMEs and PMEIs—in the reproductive tissues of these 2 species, as well as evidence that numerous of these PMPZ loci have significantly diverged between species in their expression.

### Differences in pollen-biased gene expression between male and female tissues are consistent with altered selection on pollen–pistil interactions

Among all the tissue- and species-specific patterns we observed, the most general and consistent difference was a narrowed expression domain for pollen-biased genes in *S. lycopersicum*—the selfing species—compared with a broader expression domain in the outcrossing lineage. Over 95% of pollen-biased genes with any secondary expression in style tissue were more highly expressed in *S. pennellii* styles compared with *S. lycopersicum* styles (Supplementary Table 13; Fig. 2). Of these, nearly 300 loci are not expressed in *S. lycopersicum* styles at all. This pattern was recapitulated in reproductive PMEs and PMEIs specifically, where most loci were pollen-biased and had lower style, rather than pollen, expression in the selfing species (Supplementary Tables 7 and 8).

Observing large differences in floral gene expression between species with different mating systems is not unprecedented, having been previously reported in *Arabidopsis*, *Capsella*, and *Collinsia* (Slotte *et al*. 2013; Frazee *et al*. 2021; Z. Zhang *et al*. 2022), as well as between these specific *Solanum* species (Pease *et al*. 2016a). However, of the floral expression changes attributed to stylar tissue, many affect style-biased or -exclusive functions—not, as we observe here, the stylar expression of pollen-biased genes. For instance, the transition from self-incompatibility to self-compatibility involves the mutational loss of specific stylar proteins that govern the self-incompatibility reaction (Kondo *et al*. 2002; Stone 2002; Bedinger *et al*. 2017) as has previously been reported in *S. lycopersicum* (e.g. Bedinger *et al*. 2011; Pease *et al*. 2016a) and other self-compatible species (e.g. Slotte *et al*. 2013). Style gene expression changes could also arise from floral development changes that often accompany increased selfing—especially reduced style length. The significantly shorter style of *S. lycopersicum* (Vosters *et al*. 2014) and the reduced or abolished function of specific stylar growth proteins (Pease *et al*. 2016a) have also previously been reported in this species. Unlike these effects, mating system differences in style gene expression between species have not previously been connected specifically to pollen-related functions, although İltaş *et al*. (2024) recently proposed that 25 loci differentially expressed in pollinated pistils reflected divergent maternal responses to pollination in 2 *Arabidopsis lyrata* populations with different mating systems.

What factors associated with the mating system might specifically alter the dynamics acting on the style expression of pollen-biased genes, including PMEs and PMEIs that modulate pollen tube growth? One such factor is the nature and intensity of selection acting on pollen performance traits (Mazer *et al*. 2010; Cutter 2019; and see *Introduction*). In particular, by reducing the number and diversity of pollen donors, transitions from outcrossing to selfing are expected to decrease the benefit of stylar mechanisms that amplify discrimination among alternative pollen donors (cryptic female choice). This altered selection on stylar-mediated pollen performance would be observed as reduced style-specific influence on pollen tube growth in lineages with higher selfing. This is the pattern we observe in our analysis: lower stylar expression of pollen-biased genes in *S. lycopersicum* is consistent with a reduced allocation of stylar proteins to pollen tube development and growth, compared with *S. pennellii*. Conversely, higher expression of pollen-biased loci—including PMEs and PMEIs—in the style of *S. pennellii* indicates a greater stylar contribution to modulating pollen tube growth in this outcrossing species. Variation in pollinated pistil gene expression in *Arabidopsis lyrata* populations (İltaş *et al*. 2024) has also been attributed to differential selection for cryptic female choice in selfing vs outcrossing populations. Resource reallocation is commonly observed following transitions to selfing, including shifts in investment from pollen to ovule production (as evidenced by reduced pollen–ovule ratios in more selfing species; Cruden 1977; Mione and Anderson 1992; Wozniak and Sicard 2018). Our proposal here hypothesizes a similar realignment, whereby sex allocation to stylar mechanisms of pollen tube growth is reduced in the selfing species.

### In vivo pollen performance differences are consistent with mating system effects on PMPZ pollen–pistil traits

If, as we infer, expression differences between species reflect differences in stylar contributions to pollen tube growth, these species should exhibit differential pollen performance in the presence of their own style. Consistent with this, in our in vivo assays, we observed significantly faster pollen tube growth rates in *S. pennellii* (Fig. 5), whose stylar expression of pollen tube development genes was significantly higher, compared with *S. lycopersicum* (Fig. 2). Moreover, we know this difference in pollen performance is not intrinsic to pollen (or factors like larger pollen size; e.g. McCallum and Chang 2016) alone, because the direction of differential pollen performance is reversed in the absence of stylar contributions to pollen tube development—when pollen is cultured in vitro. Under these conditions, *S. lycopersicum* pollen grows comparatively faster (Fig. 4). The specific molecular details of this differential stylar influence over pollen performance remain to be clarified. However, because the interaction of PMEs and PMEIs is known to control the equilibrium of pectin methylesterification in growing pollen tubes (Bosch and Hepler 2005; and see *Introduction*), one possibility is that the higher joint expression (across both pollen and style) of PMEs, PMEIs, and possibly other pollen growth factors enables more rapid *S. pennellii* pollen tube elongation within *S. pennellii* stylar tissue, compared with *S. lycopersicum* pollen growing in *S. lycopersicum* styles.

Under this model, our observation that *S. lycopersicum* has comparatively faster growth rates in the absence of a style (in vitro, Fig. 4) conversely suggests that its pollen might have higher intrinsic production of pollen growth factors compared with *S. pennellii*. We did not generally observe elevated pollen-biased gene expression in *S. lycopersicum* pollen but detected 1 notable exception: among the top 10 most differentially expressed pollen-biased genes (Supplementary Table 2) is 1 PME (Solyc05g054360.4) with 3-fold higher expression in *S. lycopersicum* pollen (average TPM of 5,596 vs 1,186 in *S. pennellii*; Supplementary Table 23). The biological significance of this locus for differences in species intrinsic in vitro pollen tube performance remains to be determined; however, it is intriguing to speculate that it might represent a compensatory response to the lower expression of PME genes in the *S. lycopersicum* style. Ultimately, polarizing the timing and direction of evolution of these specific expression differences between species—as well as those observed in stylar tissue—would benefit from additional gene expression data from other closely related species that also vary in their predominant mating system. Moreover, because our 2 focal species differ in many other aspects of their biology—including cultivation history, habitat differences, and abiotic climate adaptations—a broader spectrum of species comparisons would also enable us to assess the contribution of these additional factors to the patterns of gene expression and pollen performance variation we observed here.

### Implications for PMPZ trait evolution under different mating systems

Changing mating strategies can relax old constraints, and potentially impose new ones, on many traits that influence the number and identity of reproductive partners (Barrett 2002; Mazer *et al*. 2010; Cutter 2019). Our 2 focal species differ in reproductive traits that are known to be associated with mating system transitions, including changes in floral size, flower allometry (stigma exsertion), and loss of specific stylar pollen-rejection mechanisms (Covey *et al*. 2010a; Chalivendra *et al*. 2013; Vosters *et al*. 2014; Bedinger *et al*. 2017; Jewell *et al*. 2020). Our analysis here indicates that altered stylar expression of pollen-associated loci, with associated changes in the rate and stylar-independence of pollen tube growth, are also among the significant reproductive changes that differentiate these 2 species.

Our inference is that these differences are due to changes in the kind or strength of selection acting on PMPZ traits—specifically style-mediated elements of pollen–pistil interactions, as similarly inferred in İltaş *et al*. (2024). The precise eco-evolutionary conditions responsible for this observed difference remain to be definitively established. As proposed above, reduced sex allocation to stylar mechanisms of pollen tube growth could result from relaxed selection on cryptic female choice in more selfing lineages. If so, this implies that stylar-expressed pollen growth factors are normally involved in exerting this choice in outcrossing species. PME has been implicated in mate choice in the form of PMPZ reproductive isolation between maize species (Wang *et al*. 2022), so it is known to mediate stylar mechanisms of discrimination among pollen of different genotypes. Higher style expression of pollen growth genes in *S. pennellii* may enable greater fine-tuning of in vivo pollen performance, for instance, by amplifying the growth of pollen whose intrinsic expression of PMEs and PMEIs is best matched to intrinsic stylar expression of these same factors. Nonetheless, these style-mediated pollen–pistil interactions might also be shaped by several other dynamics (e.g. Harder *et al*. 2016) that could differ between outcrossing vs selfing species. For instance, under ecological conditions of strong pollen limitation, stylar promotion of pollen tube growth might better ensure the ovary receives sufficient pollen tubes to fertilize ovules; conversely, increased selfing might alleviate pollen limitation, reducing the need for stylar supplementation of pollen growth (Harder and Aizen 2010). Note that these style-mediated mechanisms need not be mutually exclusive. Promoting (compatible) pollen growth under conditions of pollen limitation and exercising cryptic female choice among pollen might be achieved by the same or similar mechanisms of stylar inputs into pollen growth.

Regardless of the specific reproductive conditions responsible, our analysis indicates that their consequences have primarily played out as changes in the gene expression profiles of styles. While other studies have also noted changes in pollination-related gene expression between mating systems using whole flower transcriptomes (Slotte *et al*. 2013; Frazee *et al*. 2021; Z. Zhang *et al*. 2022) or gene expression in pollinated pistils (İltaş *et al*. 2024), these did not localize these changes to the secondary expression of pollen-biased genes specifically in styles. Interestingly, in comparison with our detected style expression differences, evidence of systematically altered gene expression within pollen is less clear in our study. This largely “female” response is particularly intriguing, as one of its major phenotypic consequences appears to be substantial differences in male reproductive performance in the form of pollen tube growth rates. Pollen tube growth rates have direct consequences for siring success when pollen of different genotypes are competing, so rate variation is often attributed to differences in the strength of selection on pollen competition (Snow and Spira 1991, 1996; Mazer *et al*. 2010), including between closely related species that differ in the mating system (e.g. Mazer *et al*. 2018). For instance, İltaş *et al*. (2024) proposed that slower PT growth on styles of outcrossing vs selfing *A. lyrata* is due to increased stringency imposed on pollen performance, specifically by outcrossing maternal parents. Our analysis in *Solanum* instead suggests that our species differences in in vivo pollen performance are primarily the consequence of greater stylar (“female”) investment in pollen (“male”) performance in outcrossers, a pattern that would be hidden in the absence of information on style and pollen gene expression variation. Our inference underscores that a complete understanding of evolutionary variation in PMPZ traits, including “male” traits like pollen tube growth, requires the joint consideration of both male and female postmating reproductive dynamics, including how each might be shaped by changes like mating system transitions.

## Conclusions

Mating system differences between 2 *Solanum* species are associated with widespread changes in pistil gene expression, particularly of pollen-biased loci, including pollen tube development and growth genes. These differences appear to have attendant changes in pollen tube growth rates in the presence and absence of stylar factors. We infer that these species differ in style-specific control of pollen performance in vivo as a result of reduced sex allocation to stylar mechanisms of pollen tube growth in the selfing species, consistent with relaxed selection on cryptic female choice.

## Supplementary Material

jkaf107_Supplementary_Data

## Data Availability

Raw transcriptome data were obtained from NCBI SRA BioProjects PRJNA309342 (Pease *et al.* 2016a) and PRJNA245845 (Ichihashi *et al.* 2014). Data (including Supplementary tables, read counts, in vitro and in vivo images, and processed data used in R analyses), shell scripts, and R markdowns used for analyses are available on GitHub (https://github.com/biewerheisler/SolanumPenLycGeneExpressionAnalysis). Supplemental material available at G3 online.
