# Supplementary material for: Gene expression and pollen performance indicate altered postmating selection between Solanum species with different mating systems
Source: G3 (Bethesda). 2025 May 15;15(7):jkaf107. doi: 10.1093/g3journal/jkaf107 (PMC12239637; doi:10.1093/g3journal/jkaf107)
Supplement: jkaf107_Supplementary_Data [file jkaf107_supplementary_data.zip › Text_S1_G3-2025-405752.docx]

**Supplemental text to: “Gene expression and pollen performance indicate altered postmating selection between *Solanum* species with different mating systems** “

*Possible biases arising from differences between the source studies for leaf versus pollen and style transcriptomic data*

The RNAseq data used in analyses were drawn from two different experiments, which could contribute to variation in the detection of gene expression in samples from leaf (data from Ichihashi et al. 2014) versus style and pollen (data from Pease et al. 2016). However, both experiments involved similar tissue collection strategies from plants grown under greenhouse conditions. Both experiments also have high total sequencing depth in their respective tissues (>15X), indicating similar power to detect gene expression. Specifically, from the leaf experiment (Ichihashi et al. 2014), we pooled data across 7 different leaf tissues per biological replicate (whole primordia from stage 3 developing leaves and distal and proximate sections of leaf primordia from stages 4-6 developing leaves). On average, each leaf tissue had ~2.3 million reads after filtering; aggregated across 7 tissues, this is ~16.1 million leaf reads per biological replicate. From the reproductive tissue (Pease et al. 2016a), post-filtered data from pollen replicates had an average of ~43.5 million; style replicates had an average of ~36 million reads. A somewhat lower average sequencing depth for leaf tissues could potentially affect the detection of genes expressed in leaves (versus the two reproductive tissues). However, this is not borne out in the data on the total number of expressed genes detected in each tissue. Specifically, in leaves, we detected 18,516 expressed genes in at least one species, while 17829 (styles) and 7274 (pollen) loci were detected in at least one species in the two reproductive tissues (Table S1).

In addition, the structure of the main comparisons in the current study—which focus on expression differences within tissue between species and between reproductive tissues—also minimizes the potential influence of transcriptome source-study differences. This is because all tissue comparisons between species are based on paired data generated from both species in a single lab (that is, leaf data for both species comes from a single study, as does pollen and style data for both species). Similarly, all comparisons between reproductive tissues (pollen vs style) are based on data from a single study. Therefore the primary comparisons in the current analysis should not be confounded with between-study transcriptome variation.

*Robustness of results to the specific tau threshold*

In our analyses, loci with a tau value greater than or equal to 0.7 were designated as biased towards the tissue in which they were most highly expressed. This threshold for tissue-bias is common in the literature (see main text), and represents a reasonable balance between identifying loci whose expression is substantively enriched in one tissue only, without being so stringent as to exclude genes whose expression is primarily but not exclusively observed in this class of tissue. Nonetheless, we also confirmed that our major inferences are robust to a less stringent threshold for tissue-bias. To do so, we repeated all primary analyses using instead a threshold of tau>0.6. We find that the percentage of genes considered tissue-biased increases from less than a third of genes (under tau>0.7) to over 50% of all genes expressed in our dataset; the lower threshold largely adds genes that have relatively equal expression between style and leaf tissues (2159 versus 2371 pollen-biased genes, 3584 versus 5489 style-biased genes, 7968 versus 12298 leaf-biased genes, respectively at each threshold of tau>0.7 and >0.6). Using the dataset with tau>0.6, we reiterate our observations (see Results) that: species differ in the proportion of pollen-biased genes that are pollen-specific (at tau>0.6, *S. lycopersicum* has 631 specific genes out of 1227 biased loci, while *S. pennellii* has 340 specific genes out of 1144 pollen-biased genes); *S. lycopersicum* has a higher magnitude of pollen-bias (tau) across pollen-biased genes (p < 2e-16); species do not differ in the average level of expression of pollen-biased genes (p=0.901); and, secondary style expression of pollen-biased genes is significantly higher in *S. pennellii* than *S. lycopersicum* (p < 2e-16). In addition, we also reiterate that the average tau of style-biased genes does not differ between species (p=0.849) and that the average tau of leaf-biased genes is higher in *S. pennellii* than *S. lycopersicum* (p<9.64e-07). These specific analyses confirm that our two major inferences—that species express a similar number of genes but differ in the proportion of pollen-biased genes and that fewer genes are pollen-biased or -specific in the outcrossing species (Results)—are still both strongly supported at either tau threshold of 0.6 or 0.7.

Solanum *loci with previously described PMPZ functions*

For completeness, we also identified patterns of gene expression specifically in PMPZ genes involved in pollen-pistil interspecific barriers in *Solanum* (Table S9). The previously identified functions of these five loci are as follows: DIR1L (Muñoz-Sanz et al., 2021), ODC2 (Qin & Chetelat, 2021), and HT (Tovar-Mendez et al., 2014; Tovar-Mendez et al., 2016) play style-side roles in S-RNase-independent pollen rejection between self-incompatible and self-compatible species. CUL1 is involved in pollen-side unilateral interspecific incompatibility (Li & Chetelat, 2014), and FPS2 was also identified for pollen-side S-RNase-independent unilateral incompatibility (Qin et al., 2018). For at least two of these loci—OCD2 and HT—simple gene expression differences are unlikely to completely capture functional variation between species because each has only one copy in *S. lycopersicum* but greater than one known copy in *S. pennellii* (Covey et al., 2010; Qin & Chetelat, 2021) (Table S9). Nonetheless, each of these PMPZ genes had expression patterns consistent with previous analyses. In particular, both DIR1L and ODC2 were style-specific, with high expression in *S. pennellii* and low or no trace expression in *S.* lycopersicum (Table S9). DIR1L is also among the 10 most differentially expressed style-specific loci in our dataset (Table S2). CUL1 and FPS2 were both significantly more highly expressed in *S. pennellii* than *S. lycopersicum* pollen (Table S9); because both also had secondary expression in styles, they were not classified as pollen-specific or biased. HT was highly expressed and style-biased in our dataset (as previously reported in Pease et al. 2016), but stylar expression did not significantly differ between species. However, rather than gene expression variation per se, functional differences at HT between these two species are due to a mutation in the single HT copy (HT-A) in *S. lycopersicum* that results in a truncated (non-functional) protein (e.g., Covey et al. 2010). Therefore, in our analysis, each of these five PMPZ loci generated expression patterns consistent with previous characterization and, for 4 loci, with significantly higher expression in *S. pennellii* pollen or style tissues (Table S9).
